# Deubiquitinating enzymes in parkinson’s disease: molecular mechanisms and therapeutic potential

**DOI:** 10.1186/s10020-025-01389-x

**Published:** 2025-11-07

**Authors:** Yarong Wu, Yu Deng, Qi Ai, Yingzhou Li, Feiya Qin, Muzaffar Hammad, Ziyao Meng, Xiaoxia Xu, Jurui Wei, Houming Yu, Guang Liang, Xia Zhao

**Affiliations:** 1 The First People’s Hospital of Lin’an District, Affiliated Lin’an People’s Hospital, Hangzhou Medical College, Hangzhou, 310014 Zhejiang China; 2 School of Pharmacy, Hangzhou Medical College, Hangzhou, 311399 Zhejiang China; 3 Zhejiang TCM Key Laboratory of Pharmacology and Translational Research of Natural Products, Hangzhou Medical College, Hangzhou, 321399 Zhejiang China

**Keywords:** Parkinson's disease, Deubiquitinating enzymes, Physiological function, therapeutic implications, Inhibitors of dubs

## Abstract

Parkinson’s disease (PD) is a progressive neurodegenerative disorder characterized by the pathological accumulation of α-synuclein aggregates and the selective degeneration of dopaminergic neurons in the substantia nigra. Growing evidence implicates dysfunction of the ubiquitin-proteasome system (UPS), a critical regulator of protein homeostasis, in the pathogenesis of PD through impaired clearance of toxic protein species. As key components of the UPS, deubiquitinating enzymes (DUBs) counterbalance ubiquitin ligase activity by cleaving ubiquitin chains from substrate proteins, thereby playing pivotal roles in maintaining protein turnover and regulating cellular signaling pathways. Notably, emerging research has demonstrated that specific DUBs are intimately involved in modulating multiple PD-related pathological processes, including α-synuclein aggregation, mitochondrial oxidative stress, iron homeostasis, and neuronal survival. These findings suggest DUBs as promising therapeutic targets for PD intervention. This review comprehensively summarize the pathophysiological roles of PD-associated DUBs, their molecular mechanisms in disease progression, and recent advances in the development of DUB inhibitors as potential disease-modifying therapies for PD.

## Highlights

DUB dysfunction is mechanistically linked to PD pathogenesis.

DUBs integrate proteostasis imbalance and neurodegeneration by regulating α-synuclein dynamics, mitochondrial integrity, and neuronal survival.

Specific DUB modulators significantly ameliorate PD-related pathologies.

DUB-targeted strategies offer superior multi-pathway intervention compared to conventional single-target therapies for PD.

**Graphical Abstract:**

## Graphical abstract

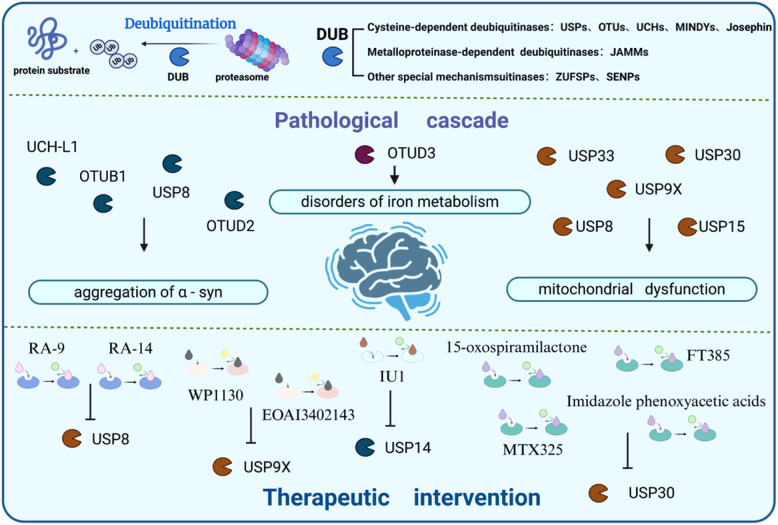

## Introduction

Parkinson’s disease (PD) is the second most common neurodegenerative disease, which seriously affects the quality of life of patients. The main pathological features of PD are the gradual loss of dopaminergic neurons and the formation of Lewy bodies composed of aggregated α-synuclein (Mehra et al. 2019; Morris et al. 2024; Geibl et al. 2024). PD is mainly treated based on symptoms, and there are no new approaches to prevent or slow the progression of the disease. Dopamine-based drugs are commonly used to treat movement disorders, of which the most widely used is the dopamine precursor levodopa (Nielsen et al. 2023; Lewis et al. 2023). The lack of a cure for PD has prompted further research into the pathogenesis of the disease and the identification of new therapeutic targets. The ubiquitin-proteasome system (UPS) is essential for the clearance of misfolded proteins, including α-synuclein (Li et al. 2022). Growing evidence indicates that UPS dysfunction contributes significantly to PD pathology by promoting the accumulation of toxic protein aggregates, ultimately leading to dopaminergic neuronal dysfunction and death (Zheng et al. 2016). This protein homeostasis disruption creates a vicious cycle wherein accumulated abnormal proteins further exacerbate neuronal injury (Domenico and Lanzillotta 2022). Therefore, targeting modulation of the UPS may be a novel strategy for the treatment of PD.

DUBs are important enzymes that regulate protein degradation and affect protein stability, localization, and function by removing ubiquitin molecules from substrate proteins (Clague et al. 2019). Accumulating evidence has established DUBs as pivotal regulators in PD pathogenesis, with their dysfunction being increasingly recognized as a key contributor to disease progression (Nielsen et al. 2023). Recent studies have shown that DUBs have a significant impact on the progression of PD by regulating neuronal survival, mitophagy, and clearance of α-synuclein (Tsefou and Ketteler 2022; Wu et al. 2024; Liang et al. 2023). For example, UCH-L1 was one of the first DUBs identified in PD and exerts a neuroprotective effect (Lee and Hsu 2017). Furthermore, OTUB1 and OTUD2 are thought to play a role in regulating inflammatory responses and mitophagy, respectively, processes that are dysregulated in PD (Xing et al. 2023; Park et al. 2023). These findings collectively suggest the therapeutic potential of targeting DUB activity to develop novel, mechanism-based interventions for PD treatment.

The development of small-molecule modulators targeting DUB activity including both inhibitors and activators represents a promising therapeutic strategy that may transcend the limitations of current dopaminergic therapies by addressing the underlying pathogenic mechanisms rather than only alleviating symptoms. This review summarizes the role and function of DUBs in PD pathogenesis, elucidates their molecular mechanisms of action, and critically evaluates recent advances in DUB-targeted pharmacological interventions. By synthesizing current knowledge on DUB biology in PD, we provide a scientific foundation for the development of novel DUB-based therapeutics that could potentially improve disease progression.

## Classification and functional characteristics of dubs

### Classification of dubs

DUBs constitute a class of proteases that catalyze the removal of ubiquitin or ubiquitin-like modifiers (including SUMO and NEDD8) from substrate proteins, thereby dynamically regulating protein stability, subcellular localization, and functional activity. These DUBs play pivotal roles in diverse cellular processes ranging from cell cycle progression and signal transduction to DNA damage repair. The human genome encodes approximately 100 functionally characterized DUBs, which can be structurally classified based on their conserved catalytic domains. These catalytic domains are frequently accompanied by various auxiliary domains that confer substrate specificity and functional regulation, including zinc finger (ZnF) domains, ubiquitin-like (UBL) folds, coiled-coil (CC) motifs, and ubiquitin-interacting motifs (UIMs) (Coleman and Huang 2018). The modular structure of DUBs, combining catalytic cores with specialized recognition domains, enables precise spatiotemporal control of ubiquitin signaling networks in response to cellular demands.

**Fig. 1:**
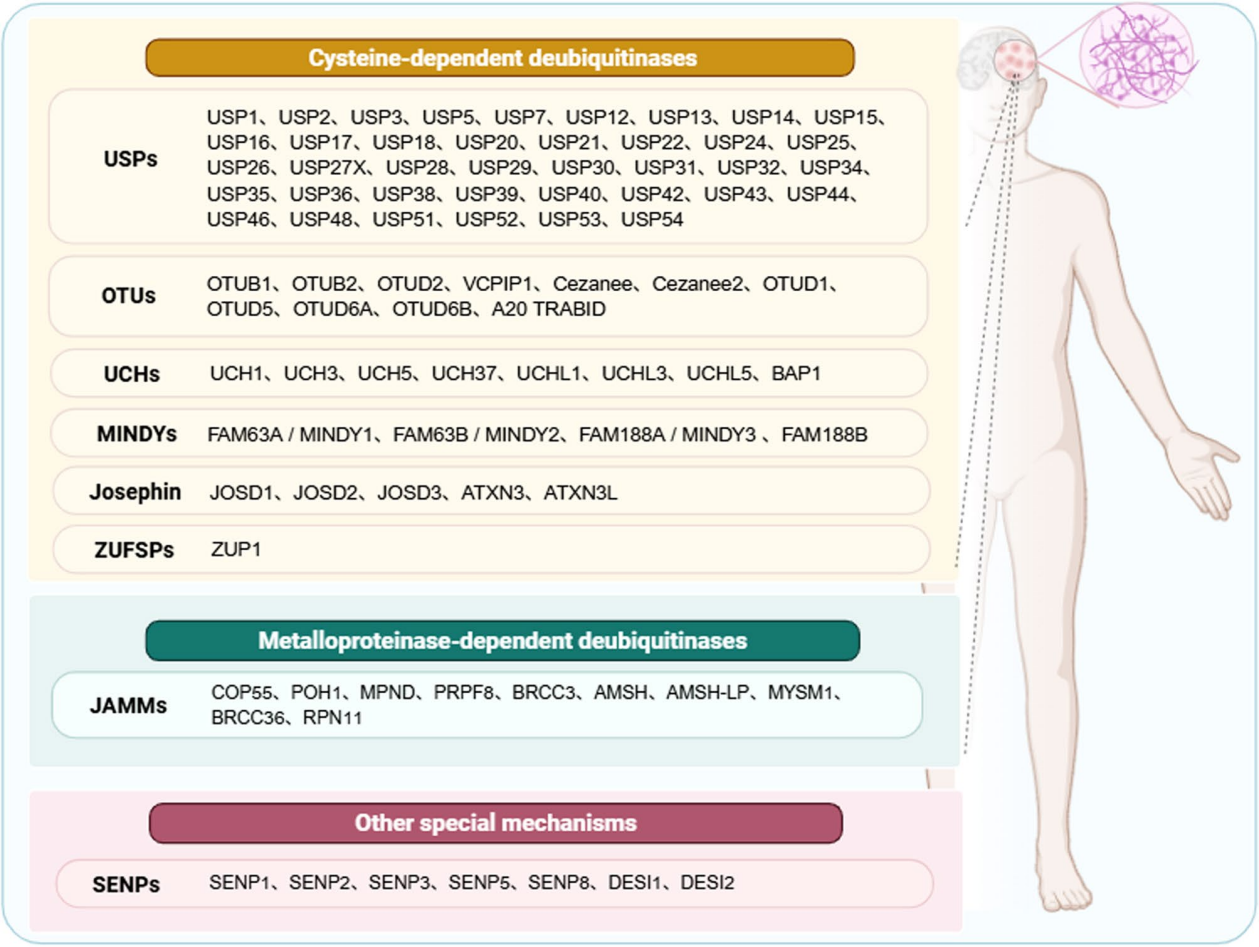
Classification of DUBs: DUBs are classified into three major categories based on their catalytic mechanisms and active site architectures: Cysteine-dependent DUBs, encompassing five structurally distinct families- ubiquitin-specific proteases (USP), ovarian tumor proteases (OTU), ubiquitin C-terminal hydrolases (UCH), Machado-Joseph disease proteases (MJD), Zinc finger-containing ubiquitin peptidase (ZUSFP)and motif interacting with ubiquitin-containing novel DUB family (MINDY) - which utilize an active-site cysteine residue for nucleophilic catalysis; Metalloprotease-dependent DUBs, represented by the JAMM/MPN + family that requires zinc ions (Zn²⁺) as essential cofactors for catalytic activity; and other mechanistically unique DUB families including SENP that employ distinctive catalytic strategies. This classification reflects the evolutionary diversity of deubiquitination machinery in eukaryotic systems

DUBs are systematically classified based on their catalytic domain architecture and mechanistic properties into several major families (Fig. 1). The predominant classes are cysteine-dependent deubiquitinases, which include five structurally distinct families: ubiquitin-specific proteases (USP), ovarian tumor proteases (OTU), ubiquitin C-terminal hydrolases (UCH), Machado-Joseph disease proteases (MJD), Zinc finger-containing ubiquitin peptidase (ZUSFP)and motif interacting with ubiquitin-containing novel DUB family (MINDY). These enzymes employ an active-site cysteine residue for nucleophilic attack during catalysis. In contrast, the JAMM/MPN family represents the sole class of zinc-dependent metalloprotease DUBs. Additionally, the recently characterized ZUFSP family exhibits a unique catalytic mechanism that distinguishes it from other DUB classes (Harrigan et al. 2018). However, the mechanisms of atypical mechanisms employed by the DUBs from the SENP families have not been elucidated.This classification reflects the evolutionary diversity of deubiquitination machinery and provides a framework for understanding their specialized functions in cellular regulation.

### Ubiquitination and deubiquitination processes

**Fig. 2:**
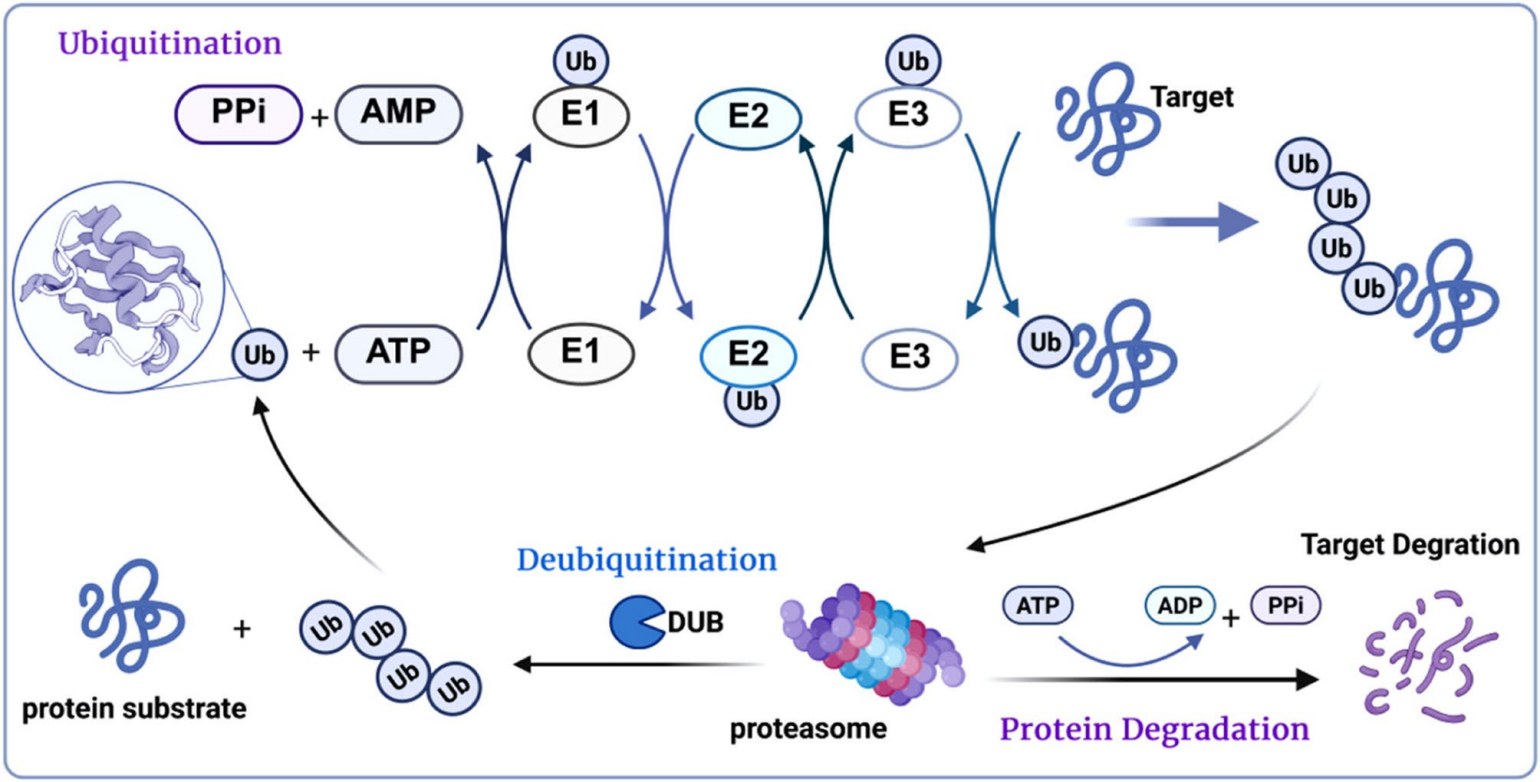
Protein ubiquitination and deubiquitination processes: The UPS operates through a precisely coordinated balance between ubiquitination and deubiquitination processes. Ubiquitination is mediated by an enzymatic cascade involving E1 (ubiquitin-activating enzyme), E2 (ubiquitin-conjugating enzyme), and E3 (ubiquitin ligase) enzymes. This process initiates with ATP-dependent ubiquitin activation by E1, followed by ubiquitin transfer to E2, and culminates in E3-mediated substrate recognition and isopeptide bond formation between ubiquitin’s C-terminus and target lysine residues on substrate proteins. The resulting polyubiquitin chains (e.g., K48-linked) serve as degradation signals for the 26 S proteasome. Conversely, DUBs counteract this process by cleaving ubiquitin-substrate or inter-ubiquitin linkages, thereby regulating substrate stability, maintaining cellular ubiquitin homeostasis, and fine-tuning ubiquitin-dependent signaling pathways. This dynamic equilibrium between ubiquitination and deubiquitination constitutes the fundamental regulatory mechanism of protein turnover in eukaryotic cells

The ubiquitination-deubiquitination cycle represents a precisely regulated, dynamic and reversible post-translational modification system that serves as the primary mechanism for controlled protein degradation in eukaryotic cells. This sophisticated process mediates the turnover of approximately 80–90% of cellular proteins (Fernández-Cruz and Reynaud 2021). Ubiquitination occurs through an enzymatic cascade involving three key components: E1 ubiquitin-activating enzymes, E2 ubiquitin-conjugating enzymes, and E3 ubiquitin ligases, which collectively mediate the ATP-dependent covalent attachment of ubiquitin molecules to specific lysine residues on target proteins (Liu et al. 2022). The reverse reaction is catalyzed by DUBs, which selectively cleave ubiquitin moieties from substrate proteins, thereby providing crucial regulation of protein stability and function (Fig. 2). This opposing enzymatic system maintains cellular protein homeostasis through continuous cycles of ubiquitin conjugation and removal, allowing for rapid responses to changing physiological conditions.

The ubiquitination system exhibits remarkable versatility through its ability to generate diverse ubiquitin chain topologies via distinct linkage types (M1, K6, K11, K27, K29, K33, K48, and K63), each mediating specific cellular functions (Akutsu et al. 2016). This structural diversity is achieved through the coordinated action of E1, E2, and E3 enzymes, which precisely assemble different chain configurations on substrate proteins. Notably, K48-linked polyubiquitin chains predominantly target substrates for proteasomal degradation, while K63-linked chains typically regulate non-proteolytic processes such as protein activity modulation and subcellular trafficking (Çetin et al. 2021). Other linkage types mediate specialized functions: K6-linked chains participate in DNA damage response pathways (Morris and Solomon 2004), K11-linked chains play crucial roles in cell cycle regulation (Bonacci and Emanuele 2019; Matsumoto et al. 2010), and K27/K33-linked chains are primarily assembled by U-BOX-type E3 ligases during cellular stress responses (Ge et al. 2023; Pawloski et al. 2022). Additionally, K29-linked chains have been implicated in the ubiquitin fusion degradation pathway (Cundiff et al. 2019; Michel et al. 2015; Tsuchiya et al. 2013). This exquisite linkage-specific regulation enables the ubiquitin system to coordinate a wide array of cellular processes with high specificity.

## PD associated dubs and their pathophysiological roles

### PD-related dubs

**Fig. 3:**
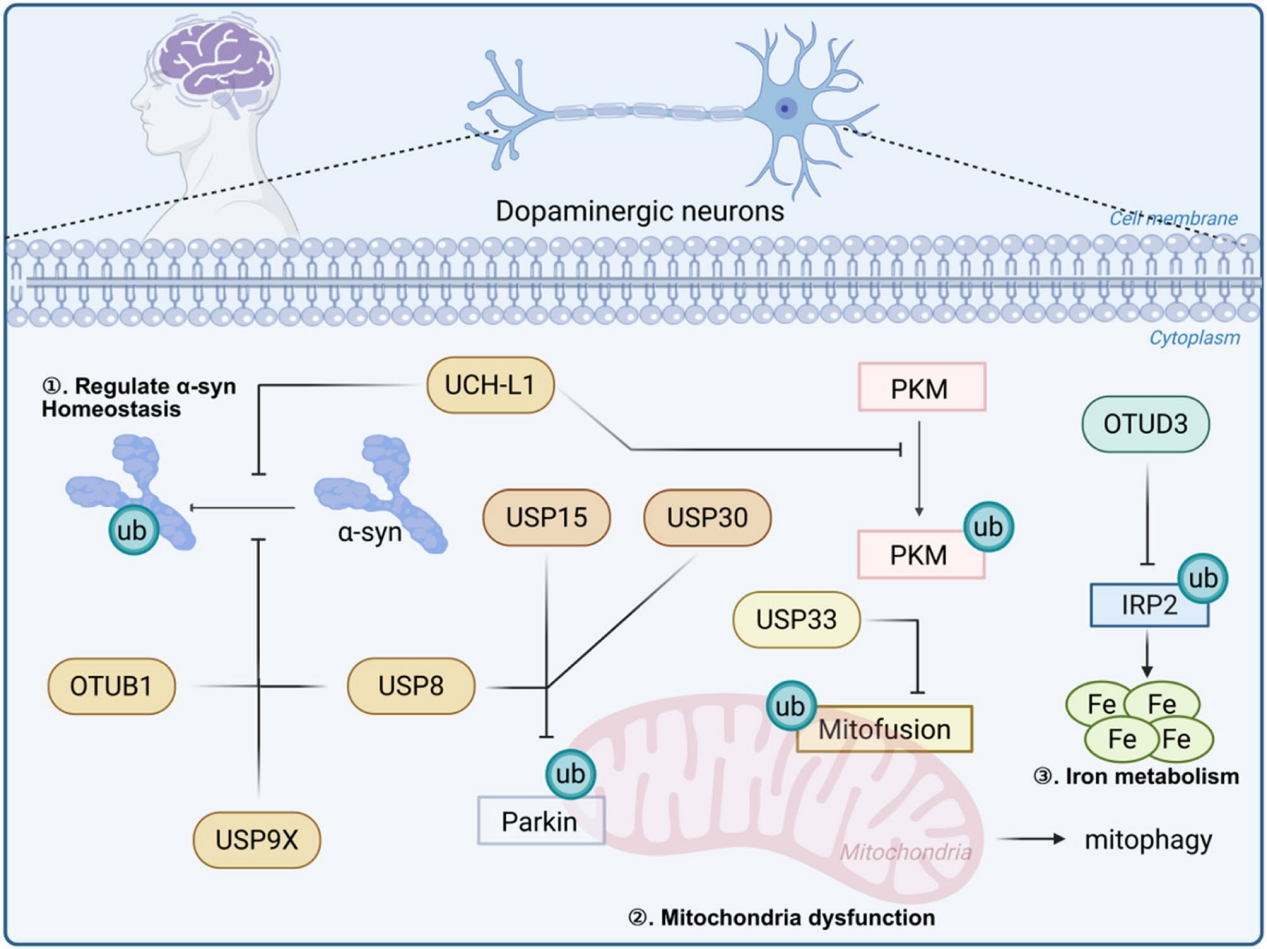
Summary of the role of DUBs in PD: The pathogenesis of PD primarily involves three interconnected pathological mechanisms: α-synuclein (α-Syn) aggregation, impaired mitophagy, and iron dyshomeostasis, with multiple deubiquitinating enzymes (DUBs) playing regulatory roles in these processes. Specifically, UCH-L1, OTUB1, USP8, and USP9X modulate α-synuclein protein stability by regulating its ubiquitination status and degradation pathways. In mitochondrial quality control, USP8, USP15, USP30, and USP33 impair Parkin-mediated mitophagy through their deubiquitination activity on mitochondrial substrates. Furthermore, OTUD3 maintains iron regulatory protein 2 (IRP2) stability independently of cellular iron status, thereby contributing to iron metabolism dysregulation observed in PD pathogenesis

DUBs have emerged as critical regulators in the pathogenesis of neurodegenerative disorders, with growing evidence implicating their dysfunction in PD pathology (Liu et al. 2023). The dynamic balance between ubiquitination and deubiquitination systems profoundly influences PD-associated neurodegenerative processes, including the formation of pathological protein aggregates and metabolic dysregulation. Therapeutic strategies targeting this balance - particularly through inhibition of specific DUBs to promote ubiquitination of pathogenic proteins and damaged organelles - show considerable promise for PD treatment (Chakraborty and Ziviani 2020). Several DUBs have been mechanistically linked to key aspects of PD pathogenesis (Table 1; Fig. 3). USP30 has been identified as a negative regulator of PINK1/Parkin-mediated mitophagy, with its overactivity leading to pathological accumulation of dysfunctional mitochondria (Bingol et al. 2014). OTUD3 plays a crucial neuroprotective role by stabilizing iron regulatory protein 2 (IRP2), thereby ameliorating iron deposition pathology in the substantia nigra (Jia et al. 2022). In line with this, hou et al. further broader significance of the OTUD3-IRP2 axis in PD iron homeostasis (Hou et al. 2024). Thus, this OTUD3-IRP2 axis represents an important mechanism governing brain iron homeostasis in PD pathophysiology. UCH-L1 demonstrates dual functionality in PD, regulating both α-synuclein degradation and exerting neuroprotective effects (Liu et al. 2002); USP15 has been shown to interfere with Parkin activity by blocking ubiquitin chain formation, consequently impairing mitochondrial quality control and autophagy processes(Cornelissen et al. 2014); These findings collectively highlight the diverse yet interconnected roles of DUBs in modulating critical pathways involved in PD development and progression.

**Table 1:** Function of dubs in PD

Classification	DUBs	Functions	Reference
Mediates α-synuclein degradation	UCH-L1	I93M mutation alters UCH-L1 conformation and produces toxicity	(Nishikawa et al. 2003)
	OTUB1	Its oligomeric and protofiber forms increase α-synuclein levels	(Kumari et al. 2020)
	OTUD2	Deubiquitinates and destabilizes α-synuclein	(Tanji et al. 2018)
	USP19	It promotes the disintegration of α-syn aggregates but inhibits their effective clearance	(Schorova et al. 2023)
	USP8	It plays a role in both Parkin-independent autophagy and stress-induced autophagy caused by mitochondrial depolarization	(Alexopoulou et al. 2016)
	USP9X	Removal of SIAH monoubiquitination of α-synuclein	(Rott et al. 2011)
Mediates mitochondrial autophagy	USP8	It plays a role in both Parkin-independent autophagy and stress-induced autophagy caused by mitochondrial depolarization	(Mauri et al. 2023)
	USP15	Counteracts Parkin-mediated ubiquitin chain formation	(Cornelissen et al. 2014)
	USP30	Counteracts Parkin-mediated ubiquitin chain formation	(Rusilowicz-Jones et al. 2020)
	USP33	Reduced stability of Parkin and its translocation to depolarized mitochondria	(Niu et al. 2020)
Mediated iron deposition	OTUD3	Regulation of ubiquitination modification of IRP2	(Bingol et al. 2014),(Hou et al. 2024)

### Regulation of α-Synuclein homeostasis by dubs in PD pathogenesis

DUBs critically influence PD progression through their direct modulation of α-synuclein ubiquitination status, thereby affecting its stability, aggregation propensity, and degradation pathways. The I93M mutation in UCH-L1 induces conformational changes that impair its deubiquitination activity and confer toxic properties, potentially contributing to PD development (Setsuie et al. 2007). Notably, proteasome inhibition studies reveal that UCH-L1 overexpression leads to microtubule-encased aggregate formation, mirroring pathological inclusion body generation in PD (Ardley et al. 2004). Mass spectrometry analyses identify OTUB1 as an α-synuclein-interacting protein, with its oligomeric and fibrillar forms elevating both total α-synuclein and pathological pS129-α-synuclein levels in SH-SY5Y cells, thereby exacerbating PD pathology (Kumari et al. 2020, 2022). Comparative gene expression profiling demonstrates upregulated OTUD2 levels in PD patient substantia nigra, with immunohistochemical studies confirming its co-localization with Lewy bodies. Mechanistically, OTUD2 directly interacts with α-synuclein to cleave K6-, K11-,K33-,K63- and K29-linked ubiquitin chains, representing a novel regulatory mechanism (Park et al. 2023). Similarly, USP8 modulates α-synuclein toxicity by reducing K63-linked polyubiquitination, with elevated expression observed in PD brains (Alexopoulou et al. 2016). USP19 emerges as a key regulator of α-synuclein’s unconventional secretion pathway, where its knockout ameliorates ubiquitinated aggregate formation (Schorova et al. 2023).USP9X orchestrates α-synuclein degradation pathway selection through deubiquitination, with its deficiency promoting toxic inclusion body formation via mono-ubiquitinated α-synuclein accumulation. The observed downregulation of USP9X in PD substantia nigra suggests therapeutic potential for combined USP9X-targeted interventions with proteasome/autophagy activators (Rott et al. 2011). These findings collectively establish DUBs as master regulators of α-synuclein proteostasis with significant implications for PD therapeutics.

### Regulation of mitochondrial homeostasis by dubs in PD pathogenesis

DUBs serve as critical modulators of mitochondrial quality control in PD, orchestrating key processes including mitophagy, mitochondrial dynamics, oxidative stress response, and energy metabolism(Park et al. 2021; Bian et al. 2024; Krassikova et al. 2021). Dysregulation of these DUB-mediated pathways contributes significantly to dopaminergic neuron degeneration (Niu et al. 2020). USP8 exemplifies this regulatory network through its direct interaction with Parkin, influencing PD pathogenesis by modulating both mitochondrial function and α-synuclein degradation pathways (Mauri et al. 2023). Notably, genetic ablation of USP8 enhances α-synuclein clearance and confers neuroprotection against α-synuclein-induced cytotoxicity in Drosophila PD models, while simultaneously promoting autophagic activity that mitigates pathological progression and neuronal apoptosis in mammalian systems (Durcan and Fon 2015). The mitochondrial DUB network demonstrates complex regulation of Parkin-mediated quality control. USP15 functionally antagonizes Parkin-dependent mitochondrial ubiquitination and subsequent autophagic clearance (Cornelissen et al. 2014), while USP30 - localized to the mitochondrial outer membrane - directly reverses Parkin-mediated ubiquitination of substrates including MFN1/2, VDAC1, and TOMM20. This deubiquitination activity effectively blocks ubiquitin signal amplification and impairs the elimination of damaged mitochondria (Rusilowicz-Jones et al. 2020). Conversely, USP33 emerges as a negative regulator of mitophagy through its deubiquitination of PRKN (Parkin). Genetic knockdown studies demonstrate that USP33 inhibition stabilizes PRKN protein levels, enhances mitophagic flux, and protects SH-SY5Y cells from MPTP-induced apoptotic death (Niu et al. 2020). These findings collectively position mitochondrial-associated DUBs as pivotal regulators of neuronal survival in PD, offering promising targets for therapeutic intervention.

### Regulation of iron metabolism by dubs in PD pathogenesis

Iron dyshomeostasis represents a hallmark feature of PD, with pathological iron deposition consistently observed in brain regions exhibiting the most prominent neurodegenerative changes (Dusek et al. 2022). The colocalization of iron with α-synuclein aggregates in the substantia nigra of PD patients suggests a potential synergistic pathological relationship, wherein iron accumulation may exacerbate dopaminergic neurodegeneration through structural and functional modulation of α-synuclein (Lu et al. 2024a, b). These observations highlight the therapeutic potential of targeting iron metabolism and ferroptosis pathways in PD treatment strategies (Lu et al. 2024a, b). Among DUBs regulating iron homeostasis, OTUD3 emerges as a critical player. OTUD3-deficient mice recapitulate key PD features including: progressive iron accumulation in the substantia nigra and striatum, motor dysfunction, and age-dependent degeneration of dopaminergic neurons (Bingol et al. 2014; Hou et al. 2024). Mechanistic studies reveal that OTUD3 regulates brain iron metabolism through stabilization of iron regulatory protein 2 (IRP2) - a master regulator of cellular iron homeostasis - in an iron-independent manner (Jia et al. 2022). Importantly, OTUD3 exerts critical neuroprotective effects in the substantia nigra by deubiquitinating and stabilizing iron regulatory protein 2 (IRP2). This mechanism functions via two key pathways: stabilized IRP2 maintains normal cellular iron homeostasis, thereby preventing pathological iron deposition and subsequent oxidative stress-a core pathological feature of PD. Notably, the patterns of iron deposition observed in PD patients and experimental models correlate with the dysregulation of specific neuroendocrine factors (e.g., leptin and cortisol). This finding suggests that neuroendocrine system dysfunction may act as an upstream driver of iron metabolism imbalance and PD progression. Therefore, the OTUD3-IRP2 axis serves as a pivotal molecular link connecting neuroendocrine disorders to downstream iron-induced toxicity in PD. These findings position the OTUD3-IRP2 axis as a promising therapeutic target for preventing iron-mediated neurodegeneration in PD, offering a novel approach to modify disease progression by maintaining proper iron homeostasis.

## Therapeutic potential of DUB-targeted strategies in PD treatment

The development of deubiquitinase-targeted therapies has emerged as a promising frontier in PD research, with two primary intervention strategies currently under investigation: small-molecule inhibitors and gene therapy approaches (Joubert 2020). Among these, small-molecule inhibitors represent the most advanced therapeutic modality, with several selective compounds demonstrating significant preclinical efficacy (Moon et al. 2021). Recent advances have yielded potent and specific inhibitors against pathogenic DUBs, including MTX325 (a USP30 inhibitor that protects against behavioral deficits and leads to increased mitophagy, decreased phospho-S129 αSyn, and attenuation of SN dopaminergic neuronal loss induced by αSyn) (Fang et al. 2023), and USP14-targeting compounds that promote mitophagy and restore mitochondrial function(Chakraborty et al. 2018). These pharmacological agents have shown considerable neuroprotective effects in various PD animal models.

**Fig. 4:**
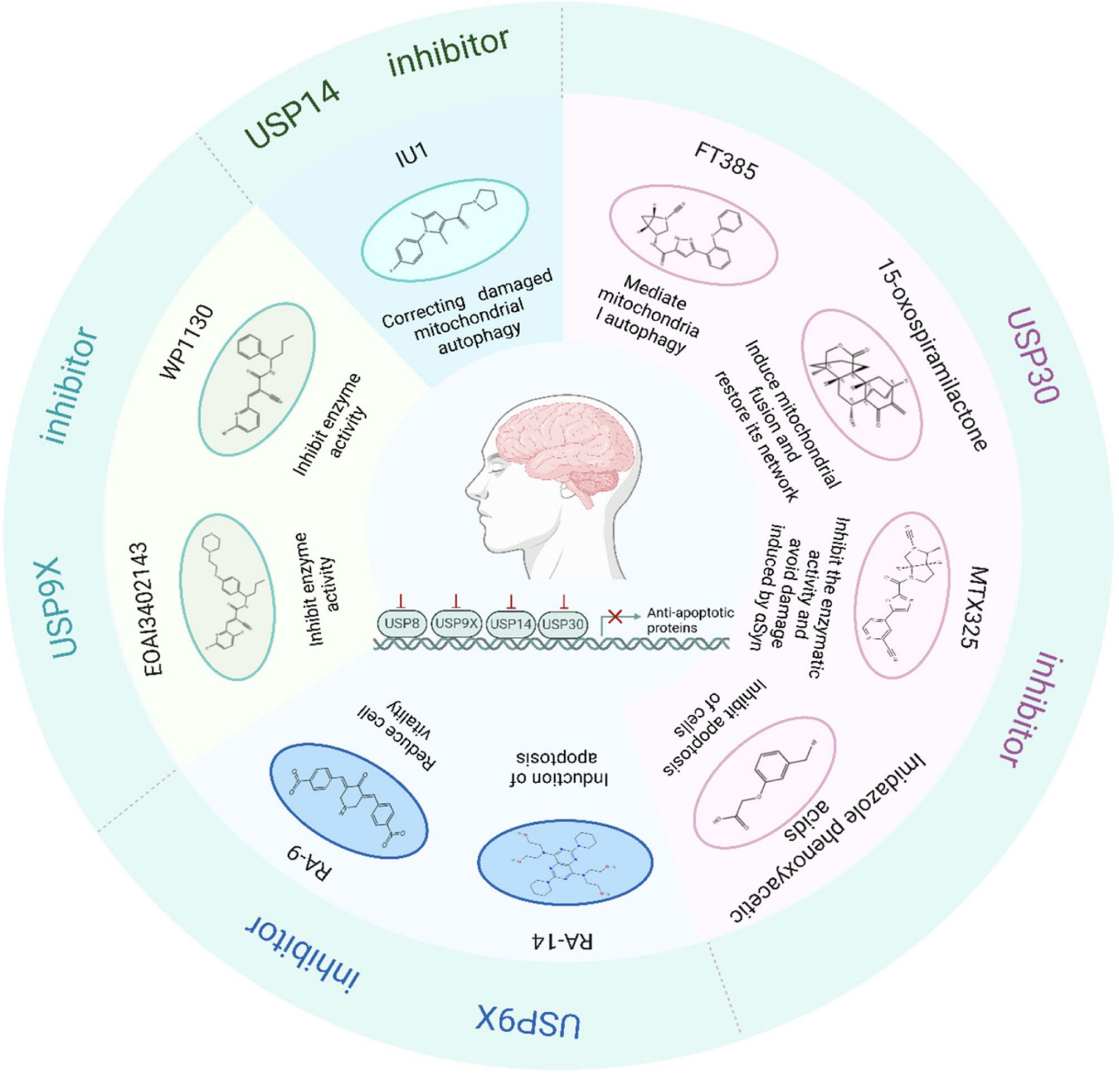
Summary of PD pathology-related DUBs inhibitor: The development of DUB inhibitors represents a promising therapeutic strategy for PD, with several candidate compounds demonstrating significant preclinical potential. Pharmacological agents targeting specific DUB families include: (Mehra et al. 2019) USP8 inhibitors RA-9 and RA-14, which modulate apoptotic pathways; (Morris et al. 2024) USP9X inhibitors WP1130 and EOAI3402149 that alter protein degradation dynamics; (Geibl et al. 2024) the USP14 inhibitor IU1, which enhances proteasome activity and mitochondrial clearance; and (Nielsen et al. 2023) USP30-targeting compounds (FT385, imidazole phenoxyacetic acids, 15-oxospiramilactone, and MTX325) that protect against α-synuclein-induced dopaminergic neurodegeneration

As previously discussed, specific DUBs contribute to PD pathogenesis through multiple mechanisms including dysregulation of α-synuclein homeostasis, impaired mitochondrial quality control, and disrupted iron metabolism (Fig. 4). While the clinical application of DUB inhibitors, designed to target conserved catalytic domains, remains in early-stage development, their therapeutic potential is substantial. Current preclinical progress in this field is summarized in Table 2, which catalogs the most promising small-molecule DUB inhibitors under investigation for PD treatment. These developments underscore the viability of DUB modulation as a disease-modifying strategy that may address multiple pathological pathways simultaneously in PD.

**Table 2:** Inhibitors of PD pathology-related dubs

Target	Inhibitor	3D-Structure	Mechanism	Reference
USP8	RA-9		Reduce cell viability	(Coughlin et al. 2014)
	RA-14		Induces apoptosis	(Issaenko and Amerik 2012)
USP9X	WP1130		Inhibition of enzyme activity	(Kapuria et al. 2010)
	EOAI3402143		Inhibition of enzyme activity	(Peterson et al. 2015)
USP14	IU1		Accelerate the degradation of wild-type tau and pathological tau mutants	(Boselli et al. 2017)
USP30	FT385		Mediates mitochondrial Function	(Rusilowicz-Jones et al. 2020)
	15-oxospiramilactone		Induces mitochondrial fusion	(Yue et al. 2014)
	MTX325		Inhibits α-synuclein induced damage	(Fang et al. 2023)
	Imidazole phenoxyacetic acids		Acts via mitochondrial clearance and cellular regeneration.	(Mandal et al. 2022)

### Therapeutic potential of USP8 inhibitors in PD

While deubiquitinase inhibitors have primarily been explored in oncology, their application to neurodegenerative diseases remains at the preclinical development stage. Issaenko et al. identified chalcone derivatives RA-9 and RA-14 as selective USP8 inhibitors with distinct mechanisms of action (Issaenko and Amerik 2012). RA-9 demonstrates potent anti-proliferative effects in cancer cells, while RA-14 induces apoptosis and significantly improves survival rates in mouse models of ES-2-induced ovarian tumors. Although these compounds have not yet been evaluated in PD models, their pharmacological targeting of USP8 holds particular relevance for PD pathogenesis. This is based on established evidence that USP8 modulates two critical PD pathways: mitochondrial function maintenance and α-synuclein homeostasis, both of which directly influence dopaminergic neuron survival. The demonstrated efficacy of these USP8 inhibitors in cancer models, combined with their specific mechanism of action, suggests strong potential for therapeutic application in PD, warranting further investigation in neurodegenerative disease models.

### Therapeutic potential of USP9X inhibitors in PD

Recent pharmacological advances have identified two potent USP9X inhibitors with distinct therapeutic profiles: WP1130, which effectively modulates USP9X deubiquitinase activity and demonstrates significant pharmacological effects (Kapuria et al. 2010), and the novel compound EOAI3402143 that exhibits dose-dependent USP9X inhibition, inducing robust tumor cell apoptosis and complete tumor regression in myeloma models (Peterson et al. 2015). Although these compounds were originally developed for oncology applications, their molecular mechanisms, particularly in regulating protein homeostasis and apoptotic pathways, show remarkable convergence with key pathological features of PD. Specifically, USP9X-mediated control of α-synuclein degradation pathways and neuronal survival mechanisms suggests these inhibitors may have direct relevance for PD treatment. The established ability of these compounds to modulate ubiquitin-dependent processes, combined with their proven blood-brain barrier permeability in preclinical models, strongly supports their potential repurposing for neurodegenerative disorders. Given these promising characteristics, systematic evaluation of USP9X inhibitors in PD models represents a critical next step for developing novel disease-modifying therapies targeting protein aggregation and neuronal vulnerability in PD.

### Therapeutic potential of USP14 inhibitors in PD management

USP14 has emerged as a critical regulator of proteostasis in PD, with its deubiquitinating activity negatively impacting both proteasomal function and mitophagy - two processes fundamentally impaired in PD pathogenesis (Kim et al. 2018). Among developing USP14 inhibitors, IU1 represents the most extensively characterized compound to date, demonstrating multiple neuroprotective mechanisms. Preclinical studies have established that IU1 facilitates the clearance of pathogenic proteins including Tau and ataxin-3, while simultaneously reducing toxic protein accumulation and subsequent neuronal damage. Notably, in genetic models of PD featuring Parkin and PINK1 mutations, IU1 administration effectively rescues mitochondrial trafficking defects and functional impairments in Drosophila models. Importantly, comprehensive toxicity assessments confirm IU1’s favorable safety profile, showing no detectable neurotoxicity at therapeutic concentrations (Boselli et al. 2017). These collective findings position IU1 and related USP14 inhibitors as particularly promising candidates for addressing the dual pathology of protein aggregation and mitochondrial dysfunction in PD. The compound’s ability to simultaneously enhance proteasomal activity and restore mitophagy suggests potential for broader therapeutic effects compared to single-pathway interventions. Further investigation is warranted to fully explore the translational potential of USP14 inhibition across different PD models and ultimately in clinical settings.

### Therapeutic potential of USP30 inhibitors in PD

Mounting evidence demonstrates that α-synuclein-induced mitochondrial dysfunction plays a pivotal role in PD pathogenesis. In chronic α-synuclein-based PD mouse models, pharmacological enhancement of mitophagy through USP30 inhibition or genetic ablation has emerged as a viable neuroprotective strategy. The USP30 inhibitor FT385, featuring a reactive cyanamide moiety, forms covalent adducts with USP30 to effectively induce mitophagy in SH-SY5Y neuronal cells(Rusilowicz-Jones et al. 2020), while imidazole phenoxyacetic acid derivatives demonstrate complementary anti-apoptotic effects in homologous cellular systems (Yue et al. 2014). INotably, impaired Mfn2 ubiquitination - a key process in mitochondrial quality control - has been directly linked to PD pathology. The compound 15-oxospiramilactone shows particular promise by fully restoring oxidative respiration capacity and mitochondrial network integrity in Mfn1/Mfn2-deficient cellular models. These findings collectively highlight USP30 inhibition as a mechanistically grounded approach to address both the mitochondrial dysfunction and impaired protein degradation pathways characteristic of PD. Continued development and optimization of these inhibitor classes may yield novel disease-modifying therapies capable of targeting multiple nodes in PD pathogenesis.

The small-molecule USP30 inhibitor MTX325 demonstrates optimal pharmacological properties for central nervous system targeting, including excellent oral bioavailability, favorable safety profile, and efficient blood-brain barrier penetration USP30 inhibition protects dopaminergic neurons in a PD model. As a physiological antagonist of PINK1/Parkin-mediated mitophagy, USP30 represents a strategic therapeutic target, with MTX325 exerting its neuroprotective effects through dual mechanisms: (Mehra et al. 2019) potent inhibition of USP30 activity and (Morris et al. 2024) enhanced ubiquitination of damaged mitochondria. Comprehensive preclinical evaluation utilizing both genetic (USP30 knockout) and pharmacological (MTX325 treatment) approaches has demonstrated consistent neuroprotection, including: (i) preservation of dopaminergic neurons and striatal dopamine levels in α-synucleinopathy models, (ii) significant reduction of pathological α-synuclein phosphorylation, and (iii) attenuation of neuroinflammatory glial activation. These multimodal therapeutic effects, observed across biochemical, cellular, and behavioral endpoints, strongly position USP30 inhibitors like MTX325 as promising disease-modifying candidates capable of addressing both the neurodegenerative and neuroinflammatory aspects of PD pathology. The convergence of favorable drug-like properties with robust efficacy in preclinical models underscores the translational potential of this therapeutic strategy.

## Discussion and future perspectives

Emerging research has established that multiple DUBs play critical roles in PD pathogenesis through their regulation of key pathological processes (Chakraborty and Ziviani 2020; Ding et al. 2024). These enzymes represent promising therapeutic targets for modulating the clearance of toxic protein aggregates in PD. However, the development of targeted therapies for DUBs is still in its infancy and faces many major challenges to be overcome. While DUBs theoretically hold promising therapeutic potential, their clinical translation faces multiple challenges. First, the specificity of DUB inhibitors remains a critical issue: The highly conserved catalytic domains among DUB family members—particularly the near-universal homology in regions adjacent to ubiquitin-binding pockets—poses significant difficulties for developing highly selective inhibitors (Harrigan et al. 2018). Initial DUB inhibitors entering clinical trials (e.g., those targeting USP7) were hindered by severe off-target effects, which not only impacted other DUB family members but also disrupted the homeostatic balance of the entire ubiquitin-proteasome system (El-Hamaky et al. 2025). Second, the blood-brain barrier (BBB) presents a major challenge in treating central nervous system disorders like PD: As the guardian of the central nervous system, the BBB’s physical barrier properties and active efflux mechanisms significantly limit the brain accumulation of over 98% of small-molecule drugs. Most DUB inhibitors are large polar molecules or contain reactive groups, severely limiting their brain exposure levels (Munavar and Lenka 2025).

Current clinical trial obstacles in DUB inhibition research remain significant. The lack of DUB inhibitors entering Phase II clinical trials primarily stems from challenges including the absence of reliable biomarkers to monitor target inhibition and pharmacodynamic effects, coupled with limited predictive power of preclinical models. Furthermore, the complex biological functions of DUBs pose toxicity risks: many DUBs (e.g., USP7) participate in multiple cellular pathways while performing seemingly contradictory roles (e.g., USP7 stabilizes p53 for tumor suppression yet promotes p53 degradation through MDM2 stabilization) (Wang et al. 2025). This functional context dependency may lead to unpredictable biological consequences when targeting specific DUBs, particularly as compensatory mechanisms and adaptive responses following prolonged inhibition could weaken therapeutic efficacy or even induce drug resistance.

Furthermore, compared to traditional inhibition strategies, emerging technologies have opened new avenues for DUB-targeted therapies, though each faces unique challenges. Take PROTAC technology as an example: By recruiting E3 ligases to induce target protein degradation, it has successfully demonstrated superior anti-tumor efficacy than conventional inhibitors in preclinical models, particularly in degrading USP7, while potentially overcoming resistance to traditional inhibitors. However, PROTAC molecules are generally bulky and exhibit poorer BBB permeability (Tashima 2023). Similarly, gene-editing technologies like CRISPR can directly correct abnormal DUB expression(Tyagi et al. 2022), but still face limitations such as low in vivo delivery efficiency, off-target editing risks, and ethical concerns, making their application in treating neurodegenerative diseases like PD unlikely in the near term.

In summary, DUBs have been demonstrated to regulate virtually all aspects of PD pathology, from protein homeostasis and mitochondrial function to neuroinflammatory responses (Buneeva and Medvedev 2024; Do and Baek 2021). While preclinical studies have validated their therapeutic potential, significant hurdles persist in inhibitor specificity and delivery optimization. The primary challenge lies in developing highly specific inhibitors that avoid interfering with critical cellular processes maintained by other functionally similar deubiquitination enzymes, which could lead to off-target toxicity. Future research directions should prioritize: (Mehra et al. 2019) development of isoform-selective inhibitors to minimize off-target effects (Morris et al. 2024), optimization of blood-brain barrier penetration, and (Geibl et al. 2024) advancement of lead compounds through clinical translation pipelines. Addressing these challenges will be essential for realizing the full therapeutic potential of DUB modulation in PD treatment and potentially other neurodegenerative disorders.We propose that future research should leverage chemical proteomics screening and computer-aided design to develop highly selective DUB modulators; explore nanocarriers, exosomes, or peptide-conjugated strategies to optimize brain delivery systems; establish reliable biomarkers; investigate combination therapy approaches; and enhance patient stratification with precision medicine.

## Data Availability

Data and materials will be made available on request.

## Abbreviations

α-syn: α-synuclein
ALP: Autophagy-lysosomal pathway
CC: Coiled-coil
CNS: Central Nervous System
DUBs: Deubiquitinating enzymes
ES-2: Extracellular Matrix Protein 2
IRP2: Iron-regulated protein 2
I93M: Isoleucine 93 - Methionine
JAMMs: JAB1/MPN domain-associated metalloproteases
Josephin: Josephin domain-containing proteases
JAMM/MPN+: JAB1/MPN/Mov34 metalloenzyme
Mfn1: Mitofusin 1
Mfn2: Mitofusin 2
MIU: Motifs interacting with the ubiquitin
MINDYs: Motif interacting with Ub-containing novel DUB family
MPTP: 1-Methyl-4-phenyl-1,2,3,6-tetrahydropyridine
MJDs: Machado-Joseph Disease protein domain proteases
NEDD8: Neural precursor cell Expressed Developmentally Down-regulated
NDs: Neurodegenerative Diseases
OTUs: Ovarian tumor structural domain proteases
OTUB1: OTU deubiquitinase, ubiquitin aldehyde binding 1
OTUD2: YOD1 deubiquitinase
OTUD3: OTU deubiquitinase 3
PD: Parkinson’s disease
PINK1: PTEN induced kinase 1
pS129: Phosphorylated Serine 129
PRKN: Parkin RBR E3 Ubiquitin Protein Ligase
SENPs: Sentrin/SUMO-specific proteases
SUMO: Small Ubiquitin-like Modifier
TOMM20: Translocase of Outer Mitochondrial Membrane 20
UPS: Ubiquitin-proteasome system
Ub: Ubiquitin
UBLs: Ubiquitin-like structural domains
UCHs: Ubiquitin C-terminal hydrolases
UCH-L1: Ubiquitin C-terminal hydrolase L1
USPs: Ubiquitin-specific proteases
USP8: Ubiquitin specific peptidase 8
USP9X: Ubiquitin specific peptidase 9 X-linked
USP15: Ubiquitin specific peptidase 15
USP25: Ubiquitin specific peptidase 30
USP30: Ubiquitin specific peptidase 30
USP33: Ubiquitin specific peptidase 33
VDAC1: Voltage-Dependent Anion Channel 1
ZnF: Zinc Finger
ZUFSPs: Zinc Finger Ubiquitin-Specific Peptidases
